# Identification of Two Maternal Transmission Ratio Distortion Loci in Pedigrees of the Framingham Heart Study

**DOI:** 10.1038/srep02147

**Published:** 2013-07-05

**Authors:** Yang Liu, Liangliang Zhang, Shuhua Xu, Landian Hu, Laurence D. Hurst, Xiangyin Kong

**Affiliations:** 1 The Key Laboratory of Stem Cell Biology, Institute of Health Sciences, Shanghai Institutes for Biological Sciences, Chinese Academy of Sciences, Shanghai 200025, China; 2 Max-Planck Independent Research Group on Population Genomics, CAS-MPG Partner Institute for Computational Biology, Shanghai Institutes for Biological Sciences, Chinese Academy of Sciences, Shanghai 200031, China; 3 Department of Biology and Biochemistry, University of Bath, Bath, UK; 4 Ruijin Hospital, Shanghai Jiaotong University School of Medicine, Shanghai 200025, China

## Abstract

Transmission ratio distortion (TRD) is indicated by the recovery of alleles in offspring in non-Mendelian proportions. An assumption of Mendelian proportion is central to many methods to identify disease-associated markers. This seems reasonable as, while TRD cases have been occasionally observed in various species few instances have been identified in humans. Here we search for evidence of paternal or maternal TRD with genome-wide SNP data of pedigrees from the Framingham Heart Study. After excluding many examples as better explained by genotyping errors we identified two maternal-specific TRD loci for autosomal SNPs rs6733122 and rs926716 (corrected *P* = 0.029 and *P* = 0.018) on *LRP2* and *ZNF133*, respectively. The transmission ratios were as high as 1.7~1.8:1. Genotyping validation and further replication is still necessary to confirm the TRD. This study shows that there may be large-effect maternal-specific TRD loci of common SNPs in the human genome but that these are rare.

Transmission ratio distortion (TRD) is indicted when the transmission ratios between different alleles deviate from the 1:1 ratio that is expected under Mendelian inheritance. TRD has been observed and studied in some model organisms, such as the segregation distorter system in *Drosophila*^1^ and the t-complex system in mouse^2^. TRD is central to understanding the fate of alleles and in the longer term TRD associated loci may be important for processes such a hybrid sterility^3^. A more immediate concern is that some genetic mapping approaches, such as sib-pair linkage analysis and transmission disequilibrium test (TDT), can be affected by TRD, since these approaches assume Mendelian inheritance^4^.

A few TRD loci have been identified in humans to date, and most of them were observed on rare variants such as the Robertsonian translocations, which were often directly associated with human disease^5^. Compared to these rare variants, TRDs on high-frequency variants such as SNPs can have more profound effects on human populations, but very few of common-variant TRD has been identified. A previous study suggested that small-effect TRDs may be very common throughout the human genome, while no large-effect TRD locus was identified^6^. Another study searched for general TRD loci with genome-wide SNP data; however, they found that most of the identified loci were false positives due to genotyping errors and it was hard to distinguish true TRD loci from the results^7^. In this study, we specifically searched for loci showing either paternal or maternal TRD with genome-wide SNP data. We robustly identify two large-effect maternal-specific TRD loci of SNPs in humans.

## Results

We used the large-scale genome-wide SNP data of individuals from the Framingham Heart Study (FHS) pedigrees (see **Methods**) to identify loci subject to paternal-specific and maternal-specific TRD. After quality-control of the data and statistical tests (see **Methods**), a total of 41 paternal and/or maternal TRD SNPs reached the 5% significance threshold after Bonferroni correction, equivalent to a raw *p*-value of 7.2 × 10^−8^ (Supplementary Table S1). After visual inspection of the cluster plots of the 41 SNPs, we found that 39 of them showed very poor genotyping quality, and the corresponding TRDs were probably caused by the misclassification of genotypes (Supplementary Fig. S1). The genotyping errors affected both paternal and maternal transmission ratios of almost all autosomal SNPs in the 39 SNPs (all paternal and maternal TRD *P* < 0.05 except for rs17628931, which gave a maternal TRD *P* = 0.056).

One of the remaining two SNPs, rs6733122, showed slightly mixed clusters of the heterozygotes and the major homozygotes (Supplementary Fig. S1). Therefore, we checked the cluster plot of its most strongly linked SNP, rs2284681 (*r*^2^ = 0.98). This SNP showed a good clustering (Supplementary Fig. S1); however, it has a less significant TRD effect (maternal TRD *P* = 2.2 × 10^−7^, corrected *P* = 0.15). Therefore, it is still possible that the TRD of rs6733122 was affected by genotyping artifact that yielded more transmissions for the major allele, and additional efforts in genotyping for this SNP would be helpful to confirm the TRD. The other remaining SNP rs926716 showed a good clustering (Supplementary Fig. S1).

The remaining two SNPs gave genome-wide-significant maternal TRD *p*-values of corrected *P* = 0.029 and *P* = 0.018 for rs6733122 and rs926716, respectively (Table 1). Interestingly, the paternal TRD *p*-values were not significant (*P* > 0.1), and we observed significant sex-differentiated TRD effects for the two identified SNPs (Table 1). In addition, assuming that the paternal TRD effects of these two SNPs existed and the transmission ratios were as small as 55%:45%, there was still 88% and 77% statistical power to detect the small paternal TRD effects of rs6733122 and rs926716, respectively, according to the total informative paternal transmission counts (352 for rs6733122 and 453 for rs926716) and the observed paternal TRD *p*-values (0.52 and 0.17). Therefore, the paternal TRD effects should be minimal and we considered that the two identified TRD loci were maternal-specific.

To avoid the situation that the TRDs were generated by a few pedigrees and to evaluate the robustness of the TRDs, we performed pedigree-based cross validation for the two maternal TRD loci (see **Methods**). The overall cross validation consistencies in the 100 repetitions of ten-fold cross validation were 953 and 962 (out of 1000) for rs6733122 and rs926716, respectively.

It is possible that the identified TRDs were confounded by actual phenotypic association if the studied individuals, especially offspring, were sampled with enrichment of certain disease (e.g., for TDT) or extreme trait, rather than randomly sampled from the population. We confirmed that the individuals of the FHS were sampled in a random way^8^,^9^,^10^. To avoid confounding by possible biased sampling towards the phenotypes of interest in the Framingham Heart Study, we also performed population-based and family-based association analyses for the two identified TRD loci with ten available phenotypes in the data (see **Methods**). Eight of the phenotypes are quantitative traits; namely, height, weight, systolic blood pressure, diastolic blood pressure, cholesterol level, high-density lipoprotein level, triglycerides level and blood sugar level, and the other two phenotypes are two disease statuses of diabetes and hard coronary heart disease. No significant association was identified (all corrected *P* > 0.05). Furthermore, we used the Genetic Association Database^11^ and GWAS Catalog^12^ to search for known disease-associated loci within the two 100-kilobase surrounding regions centered by rs6733122 and rs926716, and no disease or trait association was found.

**Figure 1:**
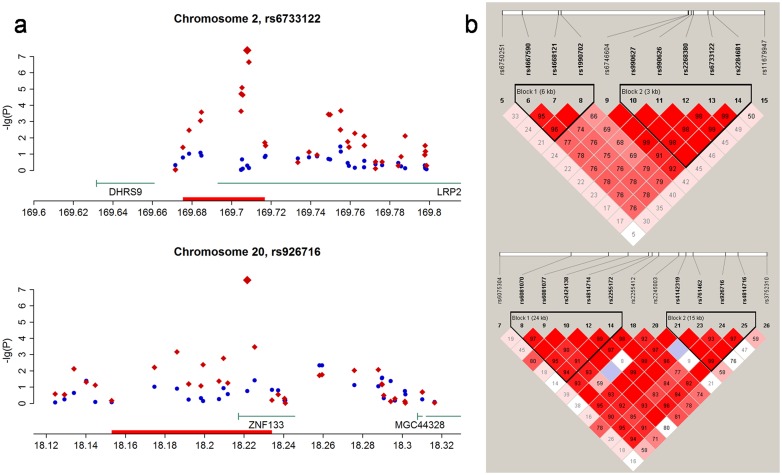
Signal plots and LD plots of two identified maternal TRD loci. (a) Regional signal plots of two identified maternal TRD loci. The TRD *p*-values of the local SNPs (within 100 kb from the identified SNP) were transformed by a negative logarithm (to base 10) and then plotted on the corresponding genomic positions (in Mb, NCBI build 36). The paternal and maternal TRD *p*-values are indicated by blue circles and red diamonds, respectively; and the enlarged diamonds in the middle denote the identified SNPs. The genes within the local region and their directions are shown below the dots. The red boxes on the position axes indicate LD regions in which the *D*' values between most of the local SNPs and the identified SNPs were larger than 0.5. (b) The LD plots of some local SNPs in the regions shown in (A) with red boxes. The LD blocks were inferred using Haploview according to the *D*' values indicated in the cells.

The first maternal TRD locus that we identified, rs6733122, is located at the 3′-end of a large gene *LRP2* (Fig. 1a), and the corresponding LD block of rs6733122 extended for 3 kb (Fig. 1b, plotted with Haploview^13^). The effect of the maternal TRD was quite large, as the transmission percentage of the major allele G was 64.6% (Table 1). *LRP2*, which encodes the megalin, is dominantly expressed in thyroid and moderately expressed in kidney (BioGPS^14^). Mutation in this gene is reported to associate with Donnai-Barrow and facio-oculo-acoustico-renal syndromes^15^, and megalin knock-out mice have high perinatal mortality owing to respiratory insufficiency^16^.

The second locus, rs926716, was located near the transcriptional start site of *ZNF133*, and the maternal TRD signal extended towards the upstream of the gene (Fig. 1). In contrast to the previous locus, the preferred maternal transmitted allele of rs926716 was the minor allele (Table 1). The TRD effect was comparable to that of the previous locus (minor allele transmission percentage 62.8%). ZNF133 is a transcription factor and it is highly expressed in CD34+ cells (BioGPS). The exact function of this gene is not clear to date.

## Discussion

After eliminating the great majority of potential incidences of TRD as most likely being genotyping errors, we identify two SNPs showing sex-specific (both maternal) TRD. A previous study also used the FHS data to search for human TRD loci of SNPs, but without separately considered for paternal and maternal TRD ref. 7. As a result, thousands of false-positive TRD loci due to genotyping error were identified and it was hard to reveal true TRD loci. In contrast, separate tests for paternal and maternal TRD can maintain the maximum power to detect paternal/maternal-specific TRD loci, and it can also help distinguish false-positive TRD loci because genotyping errors should affect both paternal and maternal transmission ratios equally. Nevertheless, the genotyping quality of the identified TRD loci is not ideal, especially for rs6733122, and genotyping validation of the SNP array data and replication in further samples from the Framingham population are still necessary to confirm the TRD.

A recent study also used the FHS data to detect general and sex-specific TRD loci in humans^17^. Excluding those probably caused by genotyping errors, no genome-wide-significant sex-specific TRD locus was identified. The two maternal TRD loci here were not identified in that study, probably because the transmissions of the ambiguous trios were included in that study for paternal or maternal transmission with a number of 0.5 for each trio as performed by PLINK, which biased the test statistic and reduced the statistical power. It has been proved that the appropriate way is to exclude these ambiguous trios for tests of paternal or maternal TRD^18^.

An early sib-pair linkage study suggested that there was the trend of extensive TRD throughout the human genome, and the trend was contributed by many small-effect TRD loci, without evidence of sex-differentiated TRD ref. 6. In this study, we showed in addition, that there are also large-effect maternal-specific TRD loci but they are rare. The TRD could be missed by linkage analysis because the markers used in linkage studies may not well capture the TRD loci^19^.

The identified maternal-specific TRD loci may result from either meiotic drive or postzygotic viability due to maternal effects (including disruptions to imprints). It is unknown what the mechanism of action might be in either of the cases that we identified. Neither marker is in a cytogenetic band with any connection to imprinting, there being no predicted imprinted genes in either 20p11 or in 2q31^20^. Nevertheless, given that megalin knock-out mice show high perinatal mortality it is worth speculating that the TRD of rs6733122 on *LRP2* is caused by postzygotic viability due to a maternal effect^16^.

The TRD loci identified in this study are probably of functional significance, and they may also relate to human diseases. Notably, rs926716 is associated with blood cholesterol level, which is an intermediate phenotype related to many complex diseases. A previous study found that a Crohn's disease-associated allele on *DLG5* is transmission-distorted among healthy male offspring^21^; and interestingly, a recent study found that a locus rs3792106 on *ATG16L1*, which is also associated with Crohn's disease, was subject to maternal TRD in healthy controls^22^. We could not replicate the maternal TRD of rs3792106 in the Framingham population (*P* = 0.70), which may be due to the fact that the TRD observed was population-specific.

In addition, we could not replicate the identified two maternal TRD loci (rs6733122 and rs926716) using two other datasets, namely, the HapMap Phase II data^23^ and the Autism Genetic Resource Exchange data^24^ (all *P* > 0.05, data not shown). Rather than declaring that the identified TRD might be statistical false positives, we speculate that large-effect TRD are likely to be observed in some specific population rather than all populations, because large-effect TRD loci are unstable, that is, the preferred alleles should quickly dominate the population after the TRD started so it is extremely unlikely that one TRD can be observed in many populations simultaneously.

Finally, it can be seen from this study that genome-wide-significant TRD loci could be identified in pedigrees without any enrichment of a certain disease in offspring, when the available sample size is large enough. It is possible that, in a TDT with genome-wide SNP data for a certain disease, there may be spurious TRD loci identified that are not necessarily relevant to the specified disease. Therefore, TDT results should be interpreted with caution, and an appropriate control for the identified TRD loci, such as to sample and test a number of unaffected trios from the studied population, would help to avoid the situation mentioned above.

## Methods

### Framingham heart study data

The data analyzed here were obtained from the GAW16 (Genetic Analysis Workshop 16) Problem 2: the Framingham Heart Study data^25^, through dbGaP^26^ (Database of Genotypes and Phenotypes, study No. phs000128, version 1). The data include ~7,000 phenotyped and genotyped individuals from three-generation pedigrees in Framingham. The SNP genotype data of Affymetrix GeneChip Human Mapping 500 K Array Set were used in this study. These genotypes were obtained from raw intensities using the genotype calling algorithm BRLMM of Affymetrix and the genotype data were directly available. No genotyping batch information is available.

### Quality control

The data mentioned above were processed with a series of quality-control procedures, as follows. (1) Removal of any individual with missing sex information; (2) removal of twins; (3) removal of any individual with a call rate < 97%; (3) exclude any SNP with > 5% missing genotypes; (4) removal of any individual with problematic sex information according to the sex inferred by the X chromosome homozygosity estimate F (male: F > 0.8; female: F < 0.2); (5) removal of out-of-pedigree singletons; (6) removal of potential duplicated samples if the estimated proportion of identical-by-decent allele sharing between any two individuals was larger than 0.9 (none removed); (7) removal of any offspring with an individual-wise Mendelian error rate > 0.01 (none removed); (8) exclude any SNP with a SNP-wise Mendelian error rate > 0.01; (9) exclude any SNP for which the proportion of "informative" (as in transmission disequilibrium test, but here we excluded ambiguous trios with all heterozygous genotypes) paternal or maternal transmissions was lower than 0.05; this is a similar control to that for minor allele frequency, but it was more appropriate in the TRD analysis to avoid sparse data; (10) remove individuals that were not in any complete parent-offspring trios. Procedures (1–6) were performed with the PLINK software^27^ and others were performed with customized programs. After these procedures, the data included 348,989 SNPs and 2,315 individuals from 319 pedigrees.

### Statistical analysis of paternal and maternal TRDs

As each child corresponds to a parental transmission event, a total of 1,241 parent-offspring trios (for 1,241 children) were derived from the 319 pedigrees for the analyses of paternal and maternal TRDs. For each SNP, the numbers of paternal and maternal allele transmissions were respectively recorded if the transmissions were informative. Transmissions in ambiguous trios with all heterozygous genotypes were not included. We used the goodness-of-fit χ^2^ test to determine if the paternal and maternal transmission ratio of two alleles of a certain SNP deviated from the expected ratio of 1:1, and used Bonferroni correction to adjust the *p*-values employing the total number of multiple tests, including the separate tests for paternal and maternal TRDs. A standard χ^2^ test was used to evaluate the difference between the paternal and maternal transmission ratio for any given SNP. The analyses were performed with customized programs and the results of the two identified TRD loci were verified with the S.A.G.E. software^28^.

### Cross validation of the TRD loci

For each identified TRD locus, ten-fold cross validation was performed and the cross validation was repeated 100 times. In each cross validation, the pedigrees were randomly divided into ten groups, and every group was labeled by turn as testing data while the other nine groups were combined and labeled as training data. In each turn, the validation was considered consistent if the direction of the TRD in the testing data was the same as that in the training data (that is, the preferred transmitted alleles in the testing data and the training data were the same).

### Phenotypic association analyses

We performed population-based phenotypic association analyses using unrelated individuals of the FHS data. Here the individuals removed in quanlity-control procedure (10) were retained because the individuals that are not in complete trios can still be used in population-based association analyses. Founders were extracted and then 444 individuals were removed due to relatedness (estimated proportion of identical-by-descent allele sharing larger than 0.125, using PLINK). A total of 1,330 individuals remained for association analyses. Eight quantitative traits and the two disease statuses were analyzed using linear regression and logistic regression, respectively, on the two identified SNPs for the evaluation of allelic additive effects. All traits except height were log-transformed to approximate normal distribution. Sex, age and smoking status were included as covariates.

Family-based association analyses were also performed using the same data as in the TRD analyses. We used QTDT^29^ to perform the association analyses of paternal and maternal allele transmissions of the two identified TRD loci with the eight quantitative traits. The same covariates as those mentioned above were included. TDT tests with the two diseases were not valid due to general TRD; therefore, we used χ^2^ tests to test for differences in allele transmission ratio between those trios with affected offspring and the other trios. Paternal and maternal allele transmission ratios were analyzed separately.

## Author Contributions

Y.L., X.K. and L.D.H. conceived and designed the experiments. Y.L. performed all the statistical and computational analyses. Y.L. and L.D.H. wrote the paper. Y.L., L.Z., S.X., L.H., L.D.H., X.K. analyzed the results and reviewed the manuscript.

## Supplementary Material

Supplementary Information Supplementary Table S1 and Figure S1

## Figures and Tables

**Table 1:** Two identified maternal TRD loci

SNP	Chr	Position	Gene	Major/minor allele (MAF)	HWE *P*	Paternal major/minor allele transmissions	Maternal major/minor allele transmissions (major allele transmission %)	Paternal TRD *P*	Maternal TRD *P* (corrected *P*)	Sex- differentiated *P*
rs6733122	2	169707961	*LRP2*	G/A (0.23)	0.89	182/170	228/125 (64.6%)	0.52	4.2 × 10^−8^ (0.029)	5.3 × 10^−4^
rs926716	20	18221632	*ZNF133*	G/C (0.37)	0.47	212/241	176/297 (37.2%)	0.17	2.6 × 10^−8^ (0.018)	3.1 × 10^−3^

The SNP positions are in NCBI build 36. Chr: chromosome. MAF: minor allele frequency. HWE: Hardy-Weinberg Equilibrium.
