# Supplementary material for: Identification of Two Maternal Transmission Ratio Distortion Loci in Pedigrees of the Framingham Heart Study
Source: Sci Rep. 2013 Jul 5;3:2147. doi: 10.1038/srep02147 (PMC3701898; doi:10.1038/srep02147)

# Supplementary Information

Identification of Two Maternal Transmission Ratio Distortion Loci in Pedigrees of the Framingham Heart Study

Yang Liu, Liangliang Zhang, Shuhua Xu, Landian Hu, Laurence D. Hurst and Xiangyin Kong

## Supplementary Table S1. 41 paternal and/or maternal TRD loci that reached the genome-wide significance threshold

| SNP | Chr | Position | Major/minor allele | Paternal major/minor allele transmissions | Maternal major/minor allele transmissions | Paternal TRD *P* | Maternal TRD *P* | Sex-differentiated *P* |
|---|---|---|---|---|---|---|---|---|
| **rs6733122** | **2** | **169707961** | **G/A** | **182/170** | **228/125** | **5.22E-01** | **4.20E-08** | **5.25E-04** |
| rs1000548 | 2 | 233900041 | T/C | 120/45 | 105/50 | 5.26E-09 | 9.98E-06 | 3.29E-01 |
| rs687813 | 3 | 76151083 | A/G | 188/94 | 239/84 | 2.17E-08 | 6.44E-18 | 4.85E-02 |
| rs7426956 | 3 | 178377531 | T/C | 280/127 | 244/136 | 3.35E-14 | 3.02E-08 | 1.73E-01 |
| rs10003632 | 4 | 150704358 | G/T | 103/3 | 99/3 | 2.66E-22 | 1.99E-21 | 9.62E-01 |
| rs17231034 | 4 | 158030994 | T/A | 94/43 | 91/26 | 1.32E-05 | 1.86E-09 | 1.02E-01 |
| rs17628931 | 4 | 171267382 | T/C | 155/70 | 121/93 | 1.46E-08 | 5.56E-02 | 7.44E-03 |
| rs4495080 | 4 | 185313697 | C/G | 238/138 | 242/137 | 2.51E-07 | 6.91E-08 | 8.74E-01 |
| rs11134178 | 5 | 6887167 | T/C | 81/33 | 90/29 | 6.94E-06 | 2.25E-08 | 4.29E-01 |
| rs16903421 | 5 | 14420568 | T/C | 150/52 | 91/33 | 5.38E-12 | 1.90E-07 | 8.62E-01 |
| rs6904469 | 6 | 17917976 | C/T | 96/43 | 91/28 | 6.94E-06 | 7.69E-09 | 1.84E-01 |
| rs9481246 | 6 | 112696203 | G/A | 277/135 | 237/121 | 2.64E-12 | 8.74E-10 | 7.62E-01 |
| rs9403326 | 6 | 141966453 | A/G | 200/160 | 203/106 | 3.50E-02 | 3.43E-08 | 7.55E-03 |
| rs609722 | 6 | 153142323 | G/C | 206/99 | 217/115 | 8.97E-10 | 2.17E-08 | 5.61E-01 |
| rs2248448 | 8 | 4528471 | C/T | 227/93 | 214/117 | 6.84E-14 | 9.74E-08 | 8.64E-02 |
| rs7024337 | 9 | 17640657 | C/G | 99/9 | 94/7 | 4.71E-18 | 4.85E-18 | 7.03E-01 |
| rs10967461 | 9 | 26648702 | C/T | 106/33 | 123/36 | 5.95E-10 | 5.22E-12 | 8.22E-01 |
| rs2399547 | 10 | 10719044 | A/G | 252/125 | 232/137 | 6.12E-11 | 7.59E-07 | 2.56E-01 |
| rs9420630 | 10 | 95518175 | G/A | 200/95 | 204/119 | 9.76E-10 | 2.25E-06 | 2.26E-01 |
| rs972676 | 11 | 17516703 | A/G | 154/300 | 171/343 | 7.28E-12 | 3.28E-14 | 8.30E-01 |
| rs10835210 | 11 | 27652486 | C/A | 174/302 | 153/295 | 4.44E-09 | 1.96E-11 | 4.45E-01 |
| rs2109185 | 12 | 5011365 | T/C | 237/152 | 247/128 | 1.63E-05 | 7.99E-10 | 1.56E-01 |
| rs12579350 | 12 | 5667362 | G/A | 114/6 | 105/7 | 6.27E-23 | 2.04E-20 | 6.79E-01 |
| rs7958718 | 12 | 60267179 | C/A | 91/58 | 136/59 | 6.86E-03 | 3.51E-08 | 9.26E-02 |
| rs11846985 | 14 | 21324445 | C/G | 229/124 | 226/122 | 2.29E-08 | 2.48E-08 | 9.85E-01 |
| rs2022818 | 14 | 27449014 | G/A | 105/8 | 82/4 | 7.17E-20 | 4.07E-17 | 4.76E-01 |
| rs1957779 | 14 | 62739400 | G/A | 180/370 | 159/334 | 5.42E-16 | 3.23E-15 | 8.70E-01 |
| rs10149025 | 14 | 94130789 | A/G | 175/65 | 168/101 | 1.24E-12 | 4.41E-05 | 1.19E-02 |
| rs12709578 | 18 | 1824649 | G/T | 252/136 | 198/112 | 3.89E-09 | 1.04E-06 | 7.68E-01 |
| rs16972328 | 18 | 35649720 | T/G | 108/3 | 89/1 | 2.14E-23 | 1.76E-20 | 4.22E-01 |

| | | | | | | | | |
|---|---|---|---|---|---|---|---|---|
| rs8099382 | 18 | 64696866 | T/G | 99/26 | 126/42 | 6.61E-11 | 9.13E-11 | 4.00E-01 |
| rs11087178 | 20 | 16553627 | C/G | 145/3 | 91/10 | 1.77E-31 | 7.64E-16 | 6.10E-03 |
| **rs926716** | **20** | **18221632** | **G/C** | **212/241** | **176/297** | **1.73E-01** | **2.64E-08** | **3.11E-03** |
| rs5997427 | 22 | 27732539 | T/C | 101/63 | 156/72 | 3.00E-03 | 2.65E-08 | 1.60E-01 |
| rs16979377 | X | 13885694 | A/G | NA | 129/0 | NA | 6.78E-30 | NA |
| rs857903 | X | 47847062 | G/A | NA | 214/59 | NA | 6.53E-21 | NA |
| rs7881759 | X | 67925498 | C/T | NA | 103/0 | NA | 3.35E-24 | NA |
| rs7066658 | X | 84734069 | A/G | NA | 104/0 | NA | 2.02E-24 | NA |
| rs12014885 | X | 117695474 | C/T | NA | 230/93 | NA | 2.48E-14 | NA |
| rs41537544 | X | 149989056 | A/G | NA | 91/0 | NA | 1.44E-21 | NA |
| rs16995982 | X | 150407618 | T/C | NA | 156/3 | NA | 7.00E-34 | NA |

The SNP positions are in NCBI build 36. Chr: chromosome. NA: not available. The two SNPs marked in bold remained after visual inspection of cluster plots.

## Supplementary Figure S1. Cluster plots

**(a)** An illustration of poor genotypic clustering for the 39 excluded SNPs. **(b)** Cluster plot of rs6733122. **(c)** Cluster plot of rs2284681, the SNP in strongest LD ($r^2$=0.98) with rs6733122. **(d)** Cluster plot of rs926716.

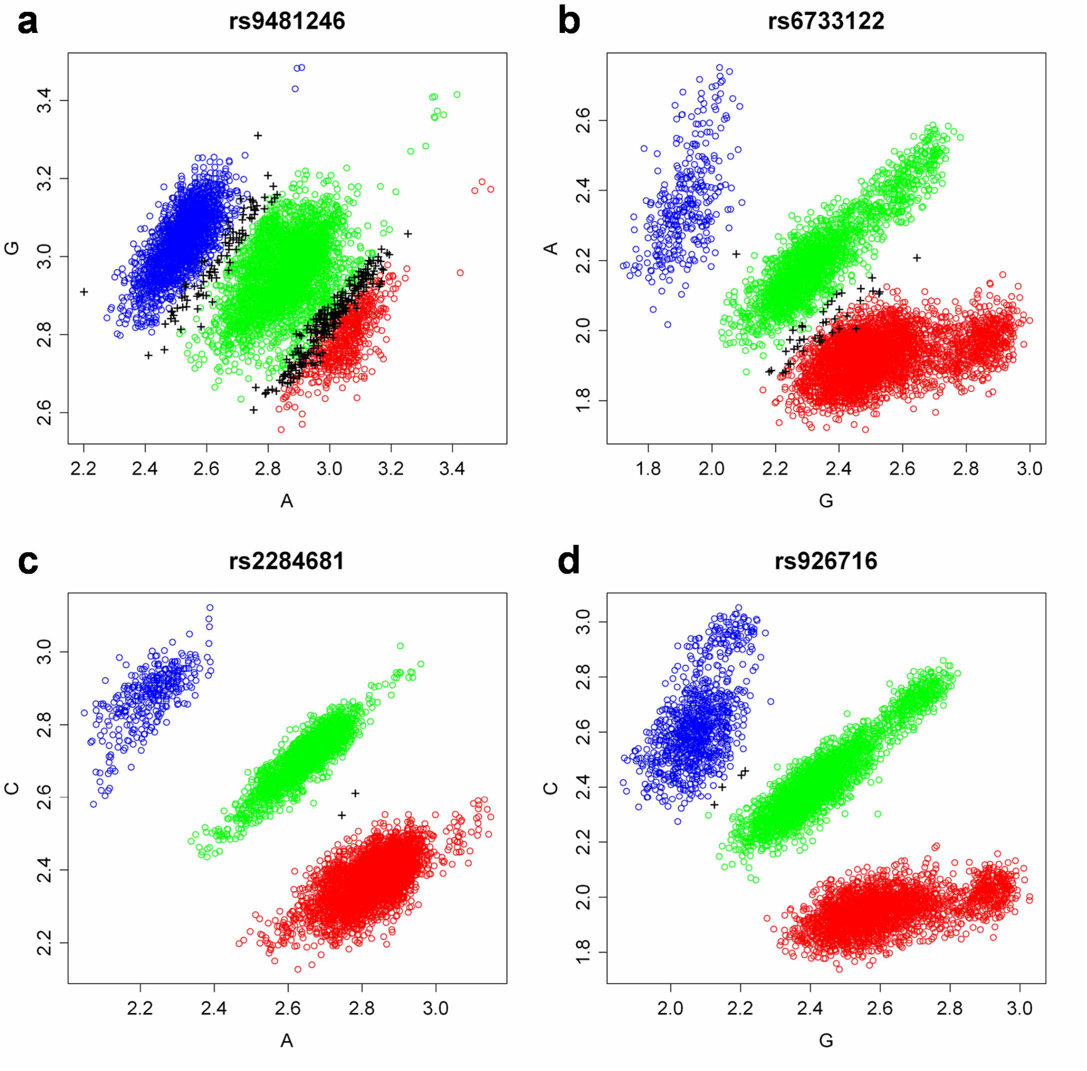

Supplement: Supplementary Information — Supplementary Table S1 and Figure S1 [file srep02147-s1.pdf]
